# Nurse-Led Microsurgical Free Flap Monitoring: A Scoping Review and Evidence-Based Framework

**DOI:** 10.3390/healthcare13212703

**Published:** 2025-10-26

**Authors:** Daihun Kang

**Affiliations:** 1 Department of Plastic and Reconstructive Surgery, Ewha Womans University Seoul Hospital, Seoul 07804, Republic of Korea; gpk1234567@naver.com; 2 College of Medicine, Ewha Womans University, Seoul 07804, Republic of Korea

**Keywords:** nursing competency, free flap monitoring, advanced nursing practice, microsurgical nursing, postoperative surveillance, nursing education, surgical nursing, scoping review

## Abstract

**Background:** Postoperative free flap monitoring, traditionally performed by surgical residents, has shifted toward nurse-led models due to global workforce constraints. While this practice is widespread, its implementation is fragmented, creating a “standardization gap” between successful outcomes and reproducible protocols. This scoping review aimed to comprehensively map all available evidence on nurse involvement in free flap monitoring and synthesize the findings into an evidence-based framework for implementation. **Methods:** A scoping review was conducted following PRISMA-ScR guidelines. A comprehensive search of PubMed, CINAHL, and the Cochrane Library was performed without date or language restrictions. Data from included studies were charted and synthesized narratively to identify key themes related to protocols, education, and clinical outcomes. **Results:** Twelve studies met the inclusion criteria. The evidence demonstrates that well-structured, nurse-led monitoring protocols achieve high flap success rates (≥94%), comparable to physician-intensive models. Three major themes emerged from the analysis: (1) the equivalence of clinical outcomes under nurse-led care; (2) the role of technology as an enabler for objective assessment and anxiety reduction; and (3) a persistent “standardization gap” due to significant variation in protocols and training across institutions. A critical knowledge deficit among nurses regarding venous congestion was also identified as a key target for educational interventions. **Conclusions:** Nurse-led free flap monitoring is a safe and effective model of care. Successful implementation hinges on a framework built upon three pillars: standardized education, clear and actionable protocols, and the standardized integration of technology. This review provides the first comprehensive roadmap to bridge the existing standardization gap and offers a foundation for developing international best-practice guidelines.

## 1. Introduction

Microsurgical free tissue transfer has revolutionized reconstructive surgery, achieving success rates exceeding 95% and restoring form and function for patients with complex defects from oncologic resection, trauma, and congenital anomalies [1]. Yet this remarkable success belies a fundamental vulnerability: the narrow window for salvage when vascular compromise occurs. Studies consistently demonstrate that while 70–80% of compromised flaps can be salvaged if detected within 24–48 h, this rate plummets to less than 30% beyond this critical period [2]. The difference between flap survival and failure often hinges not on surgical technique but on the vigilance of postoperative monitoring—making the first 72 h after surgery the most crucial determinant of ultimate success [3].

For decades, this intensive monitoring was the exclusive domain of surgical residents who maintained near-continuous bedside vigilance, performing hourly clinical assessments to detect the subtle early signs of arterial insufficiency or venous congestion [4]. This model functioned effectively when surgical training programs were robust and residents could dedicate unlimited hours to patient care. However, the traditional paradigm has been systematically dismantled by converging forces: the 2003 ACGME mandate restricting resident work hours to 80 hours per week [5], similar regulations across Europe limiting shifts to 48 h [6], and the progressive contraction of surgical training positions worldwide [7]. These pressures reached a breaking point with the 2024 mass resignation of medical residents in South Korea, where over 90% of residents abandoned their posts in protest of healthcare reforms [8], transforming major teaching hospitals into what many described as chaos—yet paradoxically, this crisis illuminated a path forward that had been slowly emerging across the global healthcare landscape.

Global workforce constraints have accelerated the adoption of nurse-led postoperative flap monitoring. The continuous bedside presence of nursing staff, combined with advances in objective monitoring technologies such as implantable Doppler systems and tissue oximetry, has enabled this transition [9,10]. Studies demonstrate that trained nurses can achieve detection rates and clinical outcomes comparable to physician-intensive models [9,11,12,13].

However, implementation has occurred in isolation, without coordination or standardization [14]. Individual institutions report excellent outcomes, yet the absence of systematic knowledge synthesis has created a “standardization gap”—a disconnect between proven success and reproducible implementation. Critical questions remain: What constitutes adequate nursing education? How can institutions balance monitoring intensity with sustainable staffing? Which technologies enhance detection versus adding complexity? How do programs successfully transition from physician-dependent to nurse-led systems? Without evidence-based guidance, institutions must independently rediscover solutions, risking patient safety. Although Varadarajan et al. (2017) demonstrated the safety of nurse-led monitoring, their focus on clinical outcomes left implementation components unmapped [14].

Therefore, this scoping review maps the current evidence on nurse-led postoperative free flap monitoring: what methods and protocols are being used, what clinical outcomes are being achieved, and what gaps exist in current practice. By identifying where standardization is lacking and what components are essential for successful implementation, this review provides a foundation for developing evidence-based approaches to nurse-led flap monitoring in the context of evolving workforce constraints.

## 2. Methods

### 2.1. Study Design

This scoping review was conducted following the Joanna Briggs Institute (JBI) methodology and is reported according to the PRISMA-ScR (Preferred Reporting Items for Systematic Reviews and Meta-Analyses Extension for Scoping Reviews) guidelines [15,16]. Unlike systematic reviews, which evaluate the effectiveness of interventions and require formal quality appraisal and risk of bias assessment, scoping reviews aim to map the breadth of available evidence and identify key concepts, gaps, and types of research in a field [16]. Accordingly, this review prioritizes methodological transparency and comprehensive evidence mapping over formal quality assessment, consistent with the exploratory and descriptive nature of scoping review methodology.

### 2.2. Research Question

The primary research question was: “What is known about nurse involvement in postoperative free flap monitoring?” This question was framed using the PCC framework to broadly capture evidence on nursing roles, protocols, education, and outcomes.

- Population: Patients undergoing free flap reconstruction
- Concept: Nurse involvement in postoperative monitoring
- Context: All surgical specialties and healthcare settings

### 2.3. Search Strategy

A comprehensive literature search was conducted from 13 September 2025 to 14 October 2025, across three electronic databases: PubMed/MEDLINE, CINAHL Plus with Full Text, and the Cochrane Library. Embase was excluded due to a lack of institutional access. No date or language restrictions were applied to ensure comprehensive coverage of the evidence base.

The search strategy was systematically developed using a combination of Medical Subject Headings (MeSH) and free-text terms with Boolean operators and truncation. The strategy was refined through iterative testing to balance sensitivity and specificity. The full PubMed search string was:

(“Free Tissue Flaps”[Mesh] OR “Surgical Flaps”[Mesh] OR “free flap*”[tiab] OR “microsurgical flap*”[tiab]) AND (nurs*[tiab] OR “Nursing Staff”[Mesh] OR “Perioperative Nursing”[Mesh]) AND (monitor*[tiab] OR “flap monitoring” [tiab] OR observ*[tiab] OR assess*[tiab] OR protocol*[tiab] OR pathway* [tiab] OR “Physiologic Monitoring”[Mesh])

Search strategies were adapted for CINAHL and Cochrane databases using their respective controlled vocabularies (CINAHL Subject Headings and Cochrane MeSH). The complete search strategies for all three databases, including adaptations of controlled vocabulary terms and keywords, are provided in Supplementary Table S1. Duplicate records were identified and removed using EndNote 21 (Clarivate Analytics, Philadelphia, PA, USA) with manual verification in Microsoft Excel. Reference lists of included articles were also manually searched to identify additional relevant studies.

### 2.4. Eligibility Criteria

To align with the broad scope of the research question, the eligibility criteria were intentionally comprehensive to capture the full range of available evidence.

#### 2.4.1. Inclusion Criteria

- Studies involving any form of nurse participation in the postoperative monitoring of free flaps;
- All study designs (e.g., experimental, observational, qualitative, mixed methods);
- All publication types (e.g., peer-reviewed articles, gray literature);
- No restrictions on language or publication year.

#### 2.4.2. Exclusion Criteria

- Studies focusing solely on surgical techniques without nursing components;
- Studies where nursing involvement could not be distinguished from other healthcare professionals;
- Conference abstracts without available full text;
- Editorials and opinion pieces without empirical data.

### 2.5. Study Selection

Two reviewers (D.K. and H.N.) independently screened titles and abstracts of all retrieved records against the eligibility criteria using a standardized screening form in Microsoft Excel. Full texts of potentially relevant articles were then independently reviewed by both reviewers against the pre-specified eligibility criteria. Results were compared at each stage, and discrepancies were resolved through discussion until consensus was reached. This dual-review process was employed to enhance the reliability of study selection, consistent with methodological rigor expected in scoping reviews. Inter-rater reliability statistics (e.g., Cohen’s κ) were not calculated, as these quantitative measures are typically applied in systematic reviews of intervention effectiveness rather than scoping reviews focused on evidence mapping [17].

### 2.6. Data Extraction

A standardized data extraction form was developed to capture relevant information from included studies (Supplementary Table S2). The form was pilot-tested on two studies to ensure comprehensiveness and clarity. The primary reviewer (D.K.) extracted data from all included studies. To ensure consistency and accuracy, the second reviewer (H.N.) independently verified all extracted data. Disagreements were resolved through discussion until consensus was achieved.

#### Extracted Data Included

- Study characteristics: Author(s), year, country, study design, sample size, setting
- Monitoring protocols: Personnel involved, monitoring frequency, nurse-to-patient ratios, assessment methods
- Clinical outcomes: Flap success rates, salvage rates, complications
- Educational components: Training programs, knowledge assessments, confidence measures
- Key findings relevant to nurse involvement

Data were organized into summary tables.

### 2.7. Data Charting and Synthesis

Consistent with scoping review methodology, no formal quality assessment of the included studies was performed. Scoping reviews are designed to map the available evidence landscape rather than synthesize effectiveness data; therefore, methodological quality appraisal and risk of bias assessment—standard for systematic reviews—are typically not conducted [15,16]. Instead, this review emphasizes comprehensive inclusion and descriptive synthesis to identify implementation components and knowledge gaps across diverse study designs and settings.

A data charting process was used to synthesize and interpret the findings. Key information from the included studies was extracted and organized into two tables.

Table 1 summarizes the general characteristics of the studies, including author, year, country, study design, and sample size.

Table 2 provides a comprehensive overview, detailing the specific monitoring protocols, reported clinical outcomes (e.g., flap success and salvage rates), and key findings from each study.

Following the data charting, a narrative synthesis was conducted to identify, analyze, and report common patterns and themes emerging from the literature. The results are presented narratively, structured around these key themes and supported by the data in the summary tables.

### 2.8. Ethical Considerations

As this review synthesized only published literature, institutional review board approval was not required. The review was conducted according to ethical standards for evidence synthesis.

## 3. Results

### 3.1. Study Selection Results

**Figure 1:**
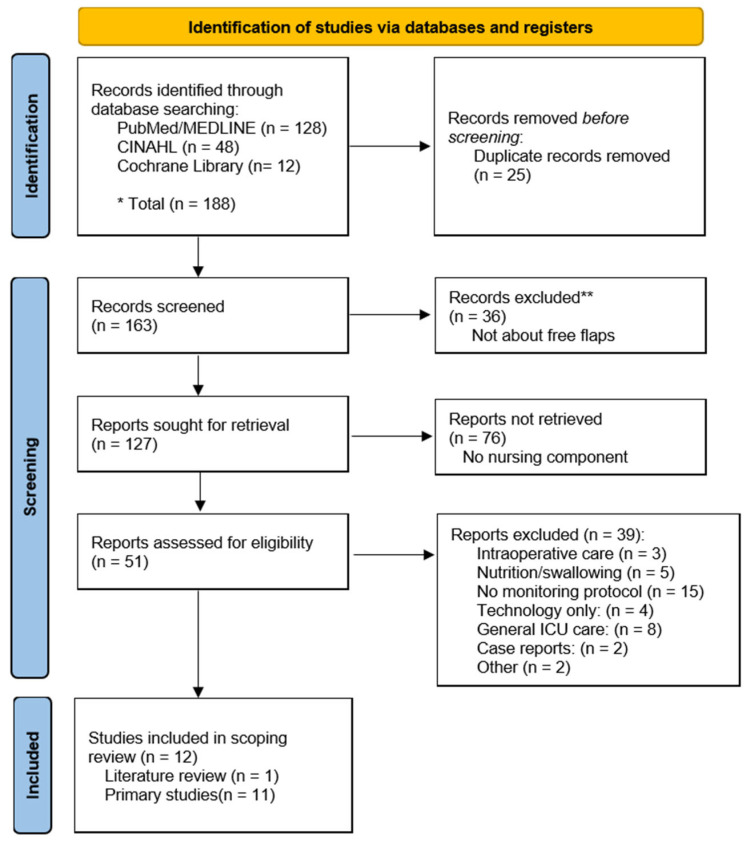
PRISMA 2020 flow diagram of the study selection process. * Total records identified across all three databases (PubMed/MEDLINE, CINAHL, and Cochrane Library) before duplicate removal. ** Records excluded during title/abstract screening as they were not related to free flap surgery.

The literature search and selection process is summarized in the PRISMA flow diagram (Figure 1). The initial search across three databases identified a total of 188 records. After 25 duplicates were removed, 163 unique records were screened based on their titles and abstracts. During this initial screening, 36 records were excluded as they were not relevant to free flap surgery.

Of the remaining 127 reports sought for full-text retrieval, a further 76 were excluded because they lacked a specific nursing component, leaving 51 articles to be assessed for eligibility. After a thorough full-text review, 39 of these articles were excluded for reasons detailed in Figure 1, including having no relevant monitoring protocol (n = 15) or focusing on general ICU care (n = 8).

Ultimately, a total of 12 studies met the inclusion criteria and were included in this scoping review. These studies consist of 11 primary research articles and one literature review. The full selection process is detailed in the PRISMA flow diagram.

### 3.2. Characteristics of Included Studies

The twelve included studies were published between 2001 and 2024 and originated from five countries: the United States (n = 8), the United Kingdom (n = 1), Spain (n = 1), Japan (n = 1), and Taiwan (n = 1). The methodologies were diverse and included retrospective studies (n = 6), a prospective cohort study (n = 1), a prospective educational intervention (n = 1), an implementation study (n = 1), a pre-post intervention study (n = 1), a quality improvement project (n = 1), and one literature review (n = 1). Sample sizes ranged from 10 flaps to 1085 flaps, with one study not specifying sample size, reflecting the diverse nature of evidence in this field (Table 1).

### 3.3. Monitoring Protocols and Implementation

Monitoring frequencies varied across institutions but demonstrated consistent patterns, with hourly nursing assessment in the initial postoperative period being the most common protocol (Table 2). Nurse-to-patient ratios, when reported, ranged from 1:2 to 1:4. Assessment methods were diverse: at least three studies reported routine use of implantable Doppler systems, one employed tissue oximetry, and two utilized a standardized monitoring chart. Many other protocols relied on clinical assessment supplemented with a handheld Doppler.

### 3.4. Clinical Outcomes

Among the ten studies that reported clinical outcomes, flap success rates were high, ranging from 94% to 100%. Most studies achieved rates at or above 95% (Table 2). Salvage rates for compromised flaps, reported in nine studies, varied more widely, from 50% to 76%. Notably, studies that directly compared different monitoring protocols or settings found no significant differences in flap loss rates. For example, there was no statistical difference in flap failure between ICU and general ward monitoring (2.9% vs. 2.6%, *p* > 0.9) or between protocols with varied resident monitoring frequency (4.2% vs. 5.9%, *p* = 0.27).

### 3.5. Educational Interventions and Nurse Competency

Four studies included an educational or competency-related component. Notably, Kleban (2020) demonstrated a significant improvement in nursing knowledge test scores from 61.9% to 89.3% after a structured educational program [9]. Other studies described educational components such as one-hour seminars (Megías Barrera, 2019 [22]) and protocol training as part of a quality improvement project (Lin, 2013 [23]), which were associated with improved process outcomes. Separately, Tsuge (2021) uniquely addressed nurse anxiety related to monitoring competency, documenting a significant reduction from 41% to 7% after implementing a user-friendly tissue oximetry device [10].

### 3.6. Emerging Themes

Three major themes emerged from the analysis:

- Equivalence of Outcomes: Nurse-led monitoring consistently achieved outcomes comparable to traditional physician-led systems across diverse settings.
- Technology as Enabler: Objective monitoring devices (tissue oximetry, implantable Doppler) reduced nurse anxiety and improved confidence while maintaining high success rates.
- Standardization Gap: Despite successful outcomes, substantial variation existed in protocols, training approaches, and documentation methods across institutions.

## 4. Discussion

### 4.1. Principal Findings

This scoping review synthesized evidence from 12 studies examining nurse involvement in postoperative free flap monitoring. The principal finding is that well-structured, nurse-led monitoring protocols achieve clinical outcomes comparable to traditional physician-intensive models, with most studies reporting flap success rates of 94% or higher. From this body of evidence, three critical themes emerged that define successful implementation: the necessity of structured education to address specific knowledge gaps, the role of technology in supporting clinical assessment and reducing psychological burden, and the persistent need for standardization across institutions.

### 4.2. Bridging the Gap in Venous Congestion Management: From Knowledge to Authorized Practice

A critical finding of this review is the substantial knowledge gap among nurses regarding venous congestion. Kleban et al. (2020) revealed that only 40.3% of nurses could correctly identify signs of venous compromise before a targeted educational intervention [9]. This is particularly alarming given that venous thrombosis is reportedly 2 to 2.5 times more common than arterial thrombosis and represents a leading cause of flap failure [9,24].

This highlights that effective education must go beyond recognizing observational signs (e.g., discoloration, brisk capillary refill) to include the competent performance and interpretation of diagnostic tests, most notably the pinprick test [25].

However, another key finding of this review is the apparent gap between the diagnostic necessity of the pinprick test and a clearly defined nursing scope of practice to perform it. For instance, the standardized monitoring charts implemented in the studies by Megías Barrera (2019) and Lin (2013), while excellent for standardizing non-invasive assessments, did not explicitly include invasive diagnostics like the pinprick test [22,23].

To translate improved nursing knowledge into improved patient outcomes, a supportive institutional framework that bridges this gap is essential. Key considerations for hospital policy should include:

- Developing explicit protocols that authorize trained nurses to perform pinprick testing.
- Providing hands-on training in both the technique and interpretation of results.
- Establishing clear escalation pathways for abnormal findings.
- Ensuring legal and institutional protection for nurses performing this expanded role.

The dramatic improvement in recognition rates (from 40.3% to 88.9%) following a brief educational intervention underscores that the identified deficit is not one of complexity, but of exposure and authorization [9]. This represents a correctable gap in nursing education and practice with a potentially significant impact on flap salvage rates.

### 4.3. Technology as a Tool for Objective Assessment

The integration of objective monitoring technologies represents a paradigm shift from subjective clinical assessment to quantifiable metrics. Among the included studies, three utilized implantable Doppler systems and one employed tissue oximetry [10,11,12,19].

The primary value lies in reducing reliance on subjective interpretation. Traditional signs like color, turgor, and capillary refill are prone to inter-observer variability, particularly during night shifts with poor lighting. Objective tools provide:

- Implantable Doppler: Real-time audible feedback on flow
- Tissue oximetry: Continuous StO_2_ values with clear thresholds

An additional benefit documented by Tsuge et al. (2021) was reduced nurse anxiety (41% to 7%) [10]. This should be viewed as a secondary outcome of having reliable data, not the primary justification.

While these objective data offer clear clinical and psychological benefits for the nursing staff, their widespread implementation faces practical hurdles. Challenges remain regarding cost, accessibility, and training requirements. Institutions must balance clinical benefits against available resources.

### 4.4. Closing the Standardization Gap: A Framework for Implementation

A central finding of this review is the paradox of success without standardization. Despite significant heterogeneity in monitoring protocols, training methods, and documentation, the included studies consistently report high flap success rates. This paradox suggests that while the core principle of nurse-led monitoring is robust, current practices operate in isolation, leaving a critical “standardization gap.” This gap is further widened by the notable absence of international guidelines or consensus statements from major microsurgical or nursing professional organizations.

To bridge this gap and translate isolated successes into a reliable, widespread standard of care, the evidence points to a framework built on three essential pillars.

#### 4.4.1. Standardized Education

A formal, structured educational program is the foundational pillar for any successful nurse-led monitoring system. This review identified that a critical knowledge deficit regarding venous congestion is a significant patient safety issue, but one that is highly correctable. As Kleban et al. (2020) demonstrated, a brief, targeted curriculum can dramatically improve nurses’ recognition rates of venous compromise [9]. This principle is reinforced by other studies where educational components—ranging from formal seminars to comprehensive quality improvement training —were integral to improving outcomes and successfully implementing new protocols. Therefore, a standardized curriculum emphasizing the clinical signs of venous versus arterial compromise, hands-on assessment techniques, and clear decision algorithms should be considered a minimum competency requirement, not an optional enhancement.

#### 4.4.2. Standardized Protocols and Tools

Effective education must be paired with clear, actionable protocols. The importance of this pillar is powerfully illustrated by foundational research from Devine et al. (2001), who found that successfully salvaged flaps were identified significantly earlier than failed flaps (mean 17.5 vs. 51 h) [21]. This provides compelling evidence that a rigorous protocol is a critical determinant of patient outcomes. This principle is reinforced throughout the included literature, where standardized protocols and tools were shown to be most effective by improving team communication, reducing detection-to-intervention times, and specifying clear, objective parameters for nursing action.

#### 4.4.3. Standardized Integration of Technology

Beyond simply adopting technology, institutions must standardize its integration into protocols. As established, objective tools like implantable Doppler systems and tissue oximetry improve outcomes and can significantly reduce nurse anxiety, a key factor in staff retention and performance. Therefore, standardization should focus on developing clear institutional guidelines for which tools to use and establishing objective, numerical thresholds for action (e.g., an StO_2_ value below 40%).

The absence of international consensus represents both a challenge and an opportunity. This scoping review, by mapping current practices and identifying these three essential pillars, provides a foundation for developing international guidelines. Professional organizations in microsurgery and nursing should collaborate to establish minimum standards, validated curricula, and best practice recommendations that can be adapted to diverse healthcare contexts while maintaining quality and safety.

### 4.5. Strengths, Limitations, and Contributions to the Field

This study’s primary strength lies in its novel contribution as the first scoping review to comprehensively map the multifaceted nature of nurse involvement in postoperative free flap monitoring. While a previous literature review by Varadarajan et al. (2017) provided crucial evidence confirming the safety and comparable efficacy of nurse-led monitoring, its focus was necessarily narrow, centered on clinical outcomes [14]. This review significantly expands upon that foundational work. The scoping methodology was deliberately chosen to move beyond the question of if nurse-led monitoring is effective, to explore how it is successfully implemented in practice. By synthesizing diverse evidence on educational frameworks, technological aids, specific protocols, and implementation challenges, this review provides, to date, the most comprehensive roadmap of the practical aspects of this evolving nursing role.

A further strength is that the scoping review methodology allowed this analysis to embrace, rather than exclude, the heterogeneity in the literature. This diversity itself revealed a critical insight: the “standardization gap.” By identifying this gap and proposing a three-pillar framework (education, protocols, technology), this review provides a foundational roadmap for developing the future best-practice guidelines that the field currently lacks.

Nevertheless, two limitations should be acknowledged. First, the exclusion of the Embase database due to institutional access limitations may have resulted in missing some relevant studies. Second, the heterogeneity that validated the choice of a scoping review also precluded a quantitative meta-analysis, a limitation inherent to the review’s broad scope and purpose.

Finally, a notable finding from mapping the literature is the scarcity of reported adverse events or negative findings, such as missed flap failures or false-positive explorations attributed to nurse-led monitoring. This absence may suggest a potential for publication bias within the field, where successful outcomes are more likely to be reported, and represents a significant knowledge gap for future research to address.

## 5. Conclusions

This scoping review confirms that nurse-led free flap monitoring is a safe and effective model of postoperative care, consistently achieving success rates exceeding 94%. The principal contribution of this review, however, is the identification of a critical “standardization gap”—a paradox where successful outcomes are achieved in isolation without reproducible, evidence-based protocols. This review concludes that bridging this gap requires an implementation framework built upon three essential pillars: (1) standardized education focused on critical knowledge deficits like venous congestion, (2) clear, actionable protocols that empower nursing staff and shorten intervention times, and (3) the standardized integration of objective technology to reduce assessment variability and psychological burden.

Implementing this three-pillar framework is not merely a recommendation for individual hospitals but a call to action for the wider professional community. Professional organizations in microsurgery and nursing should collaborate to translate these evidence-based pillars into international consensus guidelines, minimum competency standards, and validated educational curricula. As healthcare systems globally contend with workforce challenges, the evidence synthesized here provides a roadmap for the strategic expansion of nursing roles—not as a compromise, but as a robust, evidence-based advancement in patient care.

## Figures and Tables

**Table 1:** Characteristics of Included Studies.

Author (Year)	Country	Study Design	Sample Size	Setting	Primary Focus
Kleban (2020) [9]	USA	Retrospective	150 patients	Single center	ICU vs. ward
Tsuge (2021) [10]	Japan	Pre-post intervention	10 flaps	Single center	Nurse anxiety with oximetry
Anagnos (2023) [11]	USA	Prospective educational intervention	72 nurses	Single center	Nursing education
Park (2018) [12]	USA	Retrospective	122 flaps	Single center	Protocol comparison
Patel (2017) [13]	USA	Prospective cohort	1085 flaps	34 institutions	Monitoring frequency
Varadarajan (2017) [14]	USA	Literature review	17 studies	Multiple studies	Nurse vs. Physician monitoring
Stevens (2024) [18]	USA	Retrospective	803 flaps	2 hospitals	Resident monitoring frequency
Yu (2018) [19]	USA	Retrospective	512 flaps	Single center	Intermediate care unit
Jackson (2009) [20]	USA	Retrospective	94 patients	Single center	Nurse vs. resident monitoring
Devine et al., (2001) [21]	UK	Retrospective	370 flaps	Single center	Monitoring frequency
Megías Barrera (2019) [22]	Spain	Implementation study	NS	Single center	Monitoring chart
Lin (2013) [23]	Taiwan	QI project	53 patients	Single center	Protocol improvement

Abbreviations: USA = United States of America, UK = United Kingdom, ICU = Intensive Care Unit; NS = Not Specified; QI = Quality Improvement.

**Table 2:** Summary of Monitoring Protocols and Clinical Outcomes.

Author (Year)	Monitoring Protocol	Flap Success Rate	Salvage Rate	Key Findings/Other Outcomes
	(Personnel, Frequency, Tools)			
Kleban (2020) [9]	Educational Intervention	N/A	N/A	Structured education improved nurse knowledge (61.9% → 89.3%)
	(Not a clinical protocol)			
Tsuge (2021) [10]	Nurses, q4h (24–100 h), Tissue oximetry	100%	N/A	Toccare^®^ device reduced nurse anxiety (41% → 7%)
Anagnos (2023) [11]	Nurses/Residents, q1h (Nurses),	95.60%	75.5%/74.4%	No difference in failure rates with less frequent resident checks (*p* = 0.27)
	q4h vs. q12h (Residents)			
	Implantable Doppler			
Park (2018) [12]	Nurses/Residents, q1h (Nurses), q12h (Residents)	96%	64.30%	Nurse-led protocol with limited resident checks is effective and safe
	Implantable Doppler			
Patel (2017) [13]	Nurses/Residents, q1h (Nurses),	95.20%	65%	Increased frequency of resident checks does not improve outcomes
	Variable (Residents)			
	Handheld Doppler (68%)			
	Implanted Doppler (31%)			
Varadarajan (2017) [14]	Literature Review (17 studies)	95.70%	65.5% (mean)	Nurse monitoring is non-inferior to physician monitoring
Stevens (2024) [18]	Nurses, q1h, 1:4 ratio	96.70%	60%	No difference in flap loss between ICU vs. ward (*p* = 0.82)
	Clinical assessment			
Yu (2018) [19]	Nurses, q1h, 1:3 ratio	97.10%	67%	Intermediate monitoring unit is a cost-effective alternative to ICU
	Implantable Doppler			
Jackson (2009) [20]	Nurses/Residents,	94.70%	60%	Nurse-led monitoring is as effective as resident-led protocol
	q1h (Nurses), q2h (Residents)			
Devine et al., (2001) [21]	Nurses, q1h for 72 h	94%	76%	Early detection of flap compromise (avg. 17.5 h) is critical for successful salvage.
	Clinical assessment			Recommended hourly monitoring for the first 72 h.
	Doppler for buried flaps			
Megías Barrera (2019) [22]	Nurses, q1h, Standardized chart	NR	NR	Standardized chart improved communication and early detection
Lin (2013) [23]	Nurses, q1h → q4h, Clinical assessment	96.20%	50%	Protocol improvement reduced detection-to-intervention time

Abbreviations: ICU = Intensive Care Unit; N/A = Not Applicable; NR = Not Reported; q1h = every 1 h; q2h = every 2 h; q4h = every 4 h; q12h = every 12 h.

## Data Availability

No new data were created or analyzed in this study. Data sharing is not applicable to this article.

## Supplementary Materials

The following supporting information can be downloaded at https://www.mdpi.com/article/10.3390/healthcare13212703/s1, Table S1: Complete search strategies for each electronic database; Table S2: Standardized data charting form used for data extraction from included studies.
