# Supplementary material for: Nurse-Led Microsurgical Free Flap Monitoring: A Scoping Review and Evidence-Based Framework
Source: Healthcare (Basel). 2025 Oct 26;13(21):2703. doi: 10.3390/healthcare13212703 (PMC12608914; doi:10.3390/healthcare13212703)
Supplement: Supplementary file 1 [file healthcare-13-02703-s001.zip › healthcare-3906300-supplementary.pdf]

Supplementary Table S1. Complete search strategies for each electronic database.

Database	Search Strategy Details
**PubMed/MEDLINE**<br>**Date Searched:** October 14, 2025<br>**Final Results:** 128 records	("Free Tissue Flaps"[Mesh] OR "Surgical Flaps"[Mesh] OR "free flap*"[tiab] OR "microsurgical flap*"[tiab]) AND (nurs*[tiab] OR "Nursing Staff"[Mesh] OR "Perioperative Nursing"[Mesh]) AND (monitor*[tiab] OR "flap monitoring"[tiab] OR observ*[tiab] OR assess*[tiab] OR protocol*[tiab] OR pathway*[tiab] OR "Physiologic Monitoring"[Mesh])
**CINAHL Plus with Full Text**<br>**Date Searched:** October 14, 2025<br>**Final Results:** 48 records	((MH "Surgical Flaps+" OR MH "Microsurgery+") OR TI (free flap* OR microsurgical flap* OR microvascular flap*) OR AB (free flap* OR microsurgical flap* OR microvascular flap*)) AND ((MH "Nursing Care+" OR MH "Perioperative Nursing+" OR MH "Nursing Staff, Hospital+") OR TI (nurs* OR "postoperative monitoring" OR "flap monitoring") OR AB (nurs* OR "postoperative monitoring" OR "flap monitoring"))
**Cochrane Library**<br>**Date Searched:** October 14, 2025<br>**Final Results:** 12 records	#1 MeSH descriptor: [Surgical Flaps] explode all trees<br>#2 MeSH descriptor: [Microsurgery] explode all trees<br>#3 (free flap* OR free tissue transfer* OR microsurgical flap* OR microvascular flap*):ti,ab,kw<br>#4 #1 OR #2 OR #3<br>#5 MeSH descriptor: [Nursing Care] explode all trees<br>#6 MeSH descriptor: [Perioperative Nursing] explode all trees<br>#7 (nurs* OR postoperative monitoring OR flap monitoring):ti,ab,kw<br>#8 #5 OR #6 OR #7<br>#9 #4 AND #8

Notes:
MeSH = Medical Subject Headings; MH = CINAHL Heading; + = explode all subheadings; TI = Title field; AB = Abstract field; KW = Keywords field; * = truncation to capture word variations.
All searches were conducted on October 14, 2025, without date or language restrictions. Total unique records identified after deduplication: 163 (PubMed: 128, CINAHL: 48, Cochrane: 12; Duplicates removed: 25). Search strings have been iteratively refined to ensure an optimal balance between sensitivity and specificity.

Supplementary Table S2. Standardized data charting form used for data extraction from included studies.

Domain	Data Items Extracted
**STUDY IDENTIFIERS**	• First author surname<br>• Year of publication<br>• Country of origin<br>• Journal name
**STUDY CHARACTERISTICS**	• Study design<br>• Sample size<br>• Setting (single/multi-center)<br>• Care location (ICU/ward/intermediate)<br>• Surgical specialty<br>• Study period
**PATIENT/FLAP CHARACTERISTICS**	• Surgical indication<br>• Flap type and location<br>• Patient demographics (when reported)
**MONITORING PROTOCOL**	• Personnel responsible<br>• Monitoring frequency and duration<br>• Nurse-to-patient ratio<br>• Assessment methods<br>• Escalation pathways
**TECHNOLOGY INTEGRATION**	• Monitoring devices used<br>• Device specifications<br>• Threshold values for escalation<br>• Integration into workflow
**EDUCATIONAL COMPONENTS**	• Training program description<br>• Program duration and format<br>• Competency assessment methods<br>• Knowledge/skills testing<br>• Confidence measures
**CLINICAL OUTCOMES**	• Flap success rate (%)<br>• Flap failure/loss rate (%)<br>• Salvage rate for compromised flaps (%)<br>• Time to detection of compromise<br>• Complications
**IMPLEMENTATION FACTORS**	• Staffing model<br>• Institutional context<br>• Barriers and facilitators<br>• Cost considerations<br>• Protocol development process
**KEY FINDINGS**	• Main conclusions<br>• Author recommendations<br>• Study limitations noted

Notes:
This data charting form was pilot-tested on two included studies and refined before full data extraction commenced. All data extraction was performed by the primary reviewer (D.K.) with independent verification by the second reviewer (H.N.). Disagreements were resolved through discussion until consensus was achieved.
